# Patient-related outcome, fracture displacement and bone mineral density following distal radius fracture in young and older men

**DOI:** 10.1186/s12891-020-03843-9

**Published:** 2020-12-07

**Authors:** Lisa Egund, Fiona E. McGuigan, Niels Egund, Jack Besjakov, Kristina E. Åkesson

**Affiliations:** 1 Department of Clinical Sciences Malmö, Clinical and Molecular Osteoporosis Research Unit, Lund University, 205 02 Malmö, Sweden; 2 Department of Orthopedics, Skåne University Hospital, 205 02 Malmö, Sweden; 3 Department of Radiology, Aarhus University Hospital, Aarhus, Denmark; 4 Department of Radiology, Skåne University Hospital, Malmö, Sweden

**Keywords:** Distal radius fracture, Men, Function, Displacement, FRAX, BMD, patient-reported outcome

## Abstract

**Background:**

Distal radius fractures can adversely affect wrist function; for men with this fracture, the role played by fracture severity, age and osteoporosis on fracture outcome has not been sufficiently studied.

**Objective:**

To describe patient-reported outcome and the association with bone integrity, fracture severity and future fracture risk among young and older men with distal radius fracture.

**Methods:**

This prospective study includes 133 men with acute distal radius fracture, mean age 54 (range 21–88), who were followed for 12 months. They were categorized as younger (< 65) and older (65+). Main outcome was DASH (Disability of the Arm, Shoulder and Hand) at 12 months; DASH > 15 was defined as poor outcome. Fractures were classified and radiographic displacement identified at initial presentation and follow-up. BMD was measured and FRAX 10-year probability of fracture calculated.

**Results:**

Disability was higher in older men (DASH_median_ 10 vs 2; *p* = 0.002); a clinically meaningful difference (ΔDASH = 10, *p* = 0.017) remained after adjustment for displacement, fracture classification and treatment method. Almost 50% of older men vs 14% in younger had poor outcome, *p* < 0.001. Bone mineral density did not independently predict outcome. Older men with a displaced fracture at initial presentation had greater disability (DASH_median_, IQR 45, 14;73) and risk of fracture (FRAX_major osteoporotic_ 14, 8;21).

**Conclusion:**

Men over the age of 65 with a distal radius fracture are more likely to have post-fracture disability regardless of radiographic appearance. Fracture displacement, indicating impaired bone strength, is also more common and associated with an increased risk of fracture within 10-years. Secondary fracture prevention should therefore be considered in men presenting with distal radius fracture.

**Supplementary Information:**

The online version contains supplementary material available at 10.1186/s12891-020-03843-9.

## Background

A fracture of the distal radius often compromises working ability and quality of life. The distal radius fracture is already the most common fracture and incidence is increasing with the demographic shift towards an aging population [1]. The significance of the distal radius fracture lies in the fact that it is a “signal fracture” being predictive of osteoporosis and future fracture risk, particularly among post-menopausal women who also have the highest incidence of this fracture [2, 3]. Consequently, while women have been extensively studied, men are either not included or constitute a minority in most studies [4–6]. To address this paucity of studies, and hence the gaps in knowledge related to osteoporosis in males with distal radius fracture, our studies have focused solely on men. We recently showed that the prevalence of osteoporosis in this population is high, although lower compared to women [7, 8]. However, assessment of future fracture risk often is neglected in men [9].

The outcome of any osteoporosis-related fracture ranges from complete restoration of function to severe incapacitation [10–12]. These aspects are also relevant for distal radius fractures, where higher age and osteoporosis are associated with increased fracture severity and often, a higher incidence of fracture instability [4, 13–15]. For those of working age, this can mean extended time off work [16].

Traditionally, restoration of wrist function has been regarded as solely dependent on anatomic restoration of the joint. As a consequence, this has been the main ambition when treating distal radius fractures, despite the evidence being inconsistent. In older patients, some studies suggest inadequate anatomic reduction does not necessarily correspond to higher degree of disability [17–19], while others suggest that radiographic- and clinical outcomes are correlated [5, 20]. In contrast there is stronger evidence indicating that for younger individuals, fractures healed with displacement are associated with poorer outcome [5, 17, 21, 22].

For this reason, patient-perceived functional disability, may provide important complementary information, quite distinct from the radiographic outcome. Furthermore, most available data pertains to women, while it cannot be assumed to be the same for men. Therefore, the overall purpose of this investigation was to describe patient-reported outcome and the potential association with fracture severity and bone integrity in young and older men with distal radius fracture, specifically exploring the following questions:

(i) How do patient-reported outcome measures relate to radiographic parameters and is this associated with age?
(ii) The next question was, is BMD associated with fracture severity (displacement) and patient-reported outcome?
(iii) Finally, do men with distal radius fracture have a higher FRAX estimated risk of major osteoporotic fractures, and is risk associated with fracture severity and linked to patient-reported outcome?

## Methods

### Subjects and clinical protocol on admission

This was a prospective study, designed to follow patients for 12 months following a distal radius fracture, during which patient-related outcome and bone mineral density (BMD) were evaluated. Adult men from 20 years of age who presented to the Department of Orthopedics, Skåne University Hospital, Malmö, with an acute distal radius fracture were eligible for inclusion in the study. Exclusion criteria were the diagnosis of multiple fractures (including bilateral radius fracture), residency outside the hospital’s catchment area, cognitive disorder or insufficiently understanding Swedish to complete the questionnaires [7]. The specific study assessments (BMD, DASH and SF-36) were additions to the regular clinical management. This study was granted ethical approval from Lund University ethical review board and was conducted in compliance with the Helsinki Declaration. Participants provided written and informed consent at enrollment.

In total, 457 men who presented with distal radius fracture were identified and 99 were excluded (non-acute fractures 42; multiple fractures 26; died 1; non-residents 12; non-Swedish speaking 18). All the remaining 358 eligible recruits were invited to participate in the prospective study and 133 accepted and provided written and informed consent. Eight were not contactable after the initial visit to the emergency department. Reasons given for non-participation were primarily unwillingness (143, 40%) and illness (59, 16%); 15 (4%) declining participation had known active substance abuse. Non-participants did not differ from participants in terms of age distribution, however, no additional information was available for them.

All participants were assessed and managed following routine clinical practice at their initial presentation. The established treatment protocol at the clinic was evaluated in 2008 and showed good final subjective outcome [23]. Briefly, undisplaced or minimally displaced fractures were treated in a short arm cast for 4–5 weeks; displaced fractures with closed reduction and cast, and highly unstable fractures with surgery. Displacement is defined as dorsal tilt > 10° and/or ulnar variance > 2 mm. The preferred method of surgery at the time of investigation was closed reduction and external fixation. Chronological and biological age, patients’ treatment preferences and physical demands are taken into consideration. All displaced fractures were imaged at a follow-up appointment 7–10 days following the initial presentation and treatment. Thereafter, radiographs were not routine unless there were signs of a possible complication. Patients were referred to a physiotherapist who initiated rehabilitation within 1 week after removal of the cast or external, and made a final review 3–4 weeks later.

**Fig. 1:**
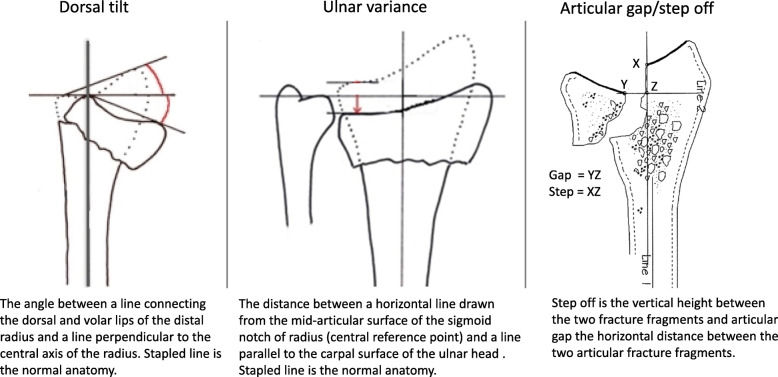
Illustration and definition of the radiographic measurements of the distal radius [24, 25]. The stapled lines demonstrate normal anatomy

At the initial presentation, standard postero-anterior (PA) and lateral radiographs were recorded [24]. If the fracture was totally undisplaced (20 patients), no further radiographic evaluation was performed. For all other participants, PA and lateral radiographs were recorded 7–10 days after the initial presentation and treatment. Two experienced radiologists (NE and JB) digitally evaluated all radiographs separately and the mean value of the two measurements was used in the analysis (Sectra IDS7 version 18.2.18.4066, Linkoping Sweden). Fractures were classified according to the AO-system: Type A - extra-articular; B - partial articular and C - complete intra-articular [18]. The severity and level of comminution was also recorded (subgroup AO type 3). The radiographic parameters measured were dorsal tilt (degrees), ulnar variance (mm), intra-articular gap and step-off (mm), see Fig. 1. Intra-observer reliability, assessed with the intraclass correlation coefficient (ICC) and 95% confidence intervals (CI) was 0.87(0.81–0.91) for ulnar variance and 0.90 (0.86–0.93) for sagittal tilt, indicating good reliability.

### Data collection and patient-related outcome measures

Participants were assessed 1 week, 6–8 weeks and 12 months after fracture. At the initial assessment a comprehensive questionnaire on health, medication and lifestyle was completed. Subsequently, the Charlson Comorbidity Index (CCI) was calculated as a measure of pre-existing comorbidity [26]. The fracture was classified as being due to a low level or high level of energy i.e. falling from less or more than a standing height, respectively. Any complication (tendon/ligament rupture, infection, carpal tunnel syndrome, complex regional pain syndrome, hardware failure etc) within the 12-month period following the fracture were determined by a retrospective chart review.

At 6–8 weeks after fracture, anthropometric characteristics were assessed at the time of bone mineral density measurement at the Osteoporosis Research unit. BMD (g/cm^2^) was assessed at the femoral neck, total hip and lumbar spine (L1-L4), using dual-energy X-ray absorptiometry (DXA), (Lunar Prodigy, GE Healthcare Lunar, Madison, Wisconsin, USA). Osteoporosis was defined as a T-score ≤ − 2.5 SD at the femoral neck, total hip and/or spine as previously reported [7, 27]. 10-year absolute fracture probability was estimated by Fracture Risk Assessment tool (FRAX®), for major osteoporotic fracture (MOF: hip, wrist, humerus and clinical spine) and for hip fracture; calculated with inclusion of femoral neck BMD [28].

Disabilities of the Arm, Shoulder and Hand (DASH) is a 30 item self-report instrument evaluating disability of the upper limb with a five likert-like response option for each item [29]. It provides a score ranging from 0 to 100 with higher scores indicating a greater level of disability. The Swedish version of the questionnaire [30] was mailed to participants at 6–8 weeks and 12 months after fracture. Pre-injury DASH score was not available. The minimum clinically relevant difference in DASH is considered to be 10 points [31].

As a measure of global health, participants completed the SF-36 health status questionnaire [32] 6–8 weeks, 12 weeks and 12 months after fracture. The instrument is compressible into the physical component score (PCS) and the mental component score (MCS), each normalized to a mean of 50 (SD 10) compared to the general population; higher scores indicating better quality of life [33].

### Statistical analysis

Categorical variables are expressed as number (%), and continuous variables as mean with standard deviation (SD) and/or range. Quantitative data were normally distributed (Kolmogorov-Smirnov test). To compare groups independent unpaired t-test was used for continuous variables and chi-square for comparisons between categorical variables (age; displacement groups). DASH data are ordinal with skewed distribution; non-parametric analyses were used and, as recommended, data is therefore presented as median (interquartile range), although mean and SD are included for comparison with published studies. To compare age and displacement we used Mann-Whitney and for multiple groups, Kruskal-Wallis.

To determine the influence of age on self-reported outcome and fracture properties, participants were grouped into: ‘younger’ below age 65 and ‘older’ 65 and above. We used this definition to facilitate comparison with the majority of published studies and because, in men, osteoporosis is more frequent from age 60–70. Adjustment was made for displacement, fracture classification and treatment method.

Inter-observer reliability of the radiographic measurements of the two radiologists was analysed using a mixed effects model with absolute agreement and reported as intraclass correlation coefficient (ICC) and 95% confidence interval (CI).

We applied multiple linear regression to determine whether BMD was predictive of patient-related outcome using continuous DASH scores at 12 months including the covariates age, treatment method, complications, radiographic parameters and CCI in the model. Although there are no validated divisions to categorize DASH scores, based on the age-specific population norm (US adult population mean DASH 10) we dichotomized DASH scores into good (< 15) or poor (≥15) outcome, to enable comparison with existing studies [5, 34]. Logistic regression analysis was used to determine if BMD is an independent predictor of poor outcome, including age, treatment method, complications, radiographic parameters and CCI. Finally, we used a generalized estimating equations analysis to explore the potential impact of femoral neck BMD and displacement at follow up on the rate of recovery in terms of DASH at 6–8 weeks and 12 months.

The final analyses included the radiographic parameters dorsal tilt and ulnar variance since exploratory analyses indicated these affected DASH negatively at 12 months, whereas intra-articulate gap and/or step-off > 1 mm did not (Supplementary Table 1). Fractures were grouped as undisplaced or displaced (dorsal tilt > 10° and/or ulnar variance > 2 mm). In 20 patients with totally undisplaced fractures and no x-ray at follow-up, we assume the fracture remained undisplaced.

Analyses were performed using SPSS v25 (IBM Corp., NY, USA). A two-tailed *p*-value < 0.05 was considered nominally significant, acknowledging that correction for multiple testing has not been performed.

## Results

In this prospective study of 133 men with distal radius fracture, mean age at fracture was 54 (Table 1). Almost two thirds of the fractures occurred after a fall from standing height. Men younger than 65, compared to those 65 years and above, had a higher proportion of fractures resulting from high trauma including falls from a height (30% vs 3%) and traffic accidents (13% vs 9%).

**Table 1:** Patient and fracture characteristics for all patients and for young and older

	All	< 65 years	≥65 years
	*n* = 133	*n* = 98	*n* = 35
Age at fracture (years)	54 ± 18 (21–88)	46 ± 14 (21–64)	75 ± 6 (67–88)
BMI (kg/m^2^)	26.0 ± 3.8 (16.0–40.8)	25.9 ± 3.8 (18.7–40.8)	26.1 ± 3.7 (16.0–31.2)
Charlson Comorbidity Index	1 ± 1 (0–6)	0 ± 1 (0–4)	2 ± 2 (0–6)
Smoking			
- current	22 (17%)	21 (21%)	1 (3%)
- former	49 (37%)	27 (28%)	22 (63%)
Medication for osteoporosis^c^	6 (5%)	2 (2%)	4 (11%)
Trauma level - low	84 (63%)	53 (54%)	31 (89%)
Dominant hand fracture	51 (38%)	35 (36%)	16 (46%)
Fracture classification AO			
A	35 (26%)	21 (21%)	14 (40%)
B	13 (19%)	12 (12%)	1 (3%)
C	76 (57%)	57 (58%)	19 (56%)
Displacement^b^			
Initial	46 (39%)	31 (35%)	15 (50%)
Follow-up	21 (23%)	7 (8%)	14 (41%)
Treatment			
Cast	68 (51%)	49 (50%)	19 (54%)
Closed reduction & cast	31 (23%)	23 (23%)	8 (23%)
Surgery	34 (26%)	26 (27%)	8 (23%)
BMD femoral neck (g/cm2)	0.929 ± 0.14 (0.57–1.27)	0.958 ± 0.14 (0.63–1.27)	0.851 ± 0.12 (0.53–1.10)
Osteoporosis^a^	24 (18%)	15 (15%)	9 (26%)
FRAX_BMD_ (median, IQR)^d^			
10-year risk of MOF	6.6 (4.5;10)	5.0 (3.9;9.4)	8.8 (6.8;14.8)
10-year risk of Hip fracture	1.6 (0.6;3.9)	0.7 (0.4;1.7)	3.6 (2;7.7)

Age, height, weight, BMI and BMD are reported as mean (SD) and range; other parameters are reported as number (%), ^a^T-score ≤ −2.5 at femoral neck, total hip or spine, ^b^Displacement: Dorsal tilt > 10° and/or ulnar variance > 2 mm °, ^c^Calcium/Vitamin D, bisphosphonates, ^d^FRAX calculated for cases from the age of 40 years. The total numbers may vary slightly because of missing data

**Fig. 2:**
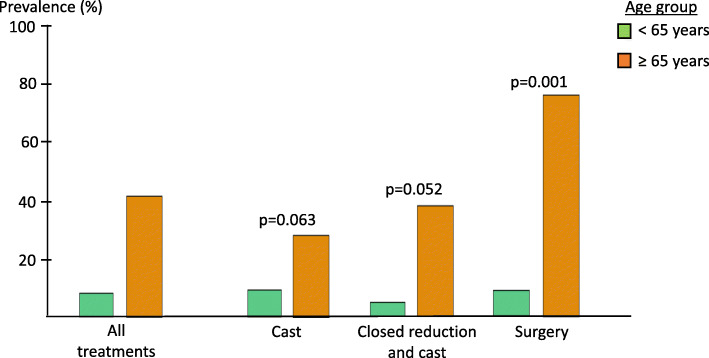
Proportion of younger and older men with displaced fractures at follow up according to type of treatment

The data shows that older men had a tendency towards a higher proportion of AO type A fractures, (Table 1), but the proportion of comminuted fractures (subgroup AO type 3) was similar in both age-groups. A higher proportion of older men had displaced fractures, particularly at follow-up (14/34 compared to 7/87 in younger men, *p* < 0.001) and this was irrespective of treatment method (Fig. 2). Complications were recorded in 12 patients (3 cast/9 surgery): 3 tendon ruptures, 4 pin infections, 1 internal implant loosening, 1 developed chronic regional pain syndrome, 1 scapholunate ligament rupture and 2 had secondary surgery due to symptomatic late displacement. Complications was similar in both age groups, 7 had secondary surgery due to displacement at follow up, of whom 2 were older than 65.

### Patient-related outcome measures - age and fracture characteristics

**Fig. 3:**
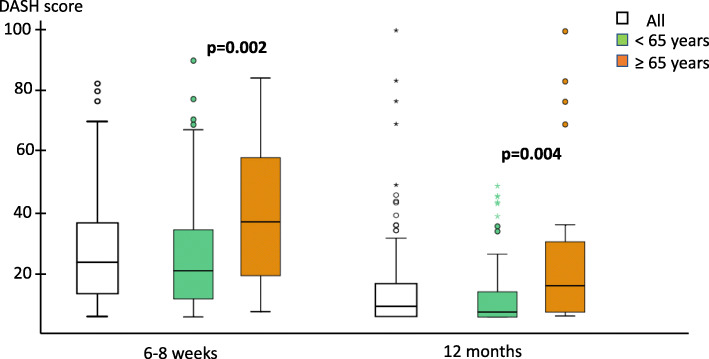
Self-reported outcome (DASH score) at 6–8 weeks and 12 months for the entire cohort and younger and older men. The boxes represent median and interquartile range

Age influenced disability at both 6–8 weeks and 12 months, although DASH scores improved over time regardless (Fig. 3). In men 65 and above, DASH at 12 months was higher (median 10 (IQR:1; 26); mean 20) compared to younger men (median 2 (IQR:0; 8); mean 7); *p* = 0.004. The difference remained after adjustment (ΔDASH 10 [95% CI 2–19], *p* = 0.017). Poor outcome as defined by DASH > 15 was subsequently also more frequent in older men (13/27 vs 11/78, *p* < 0.001).

**Fig. 4:**
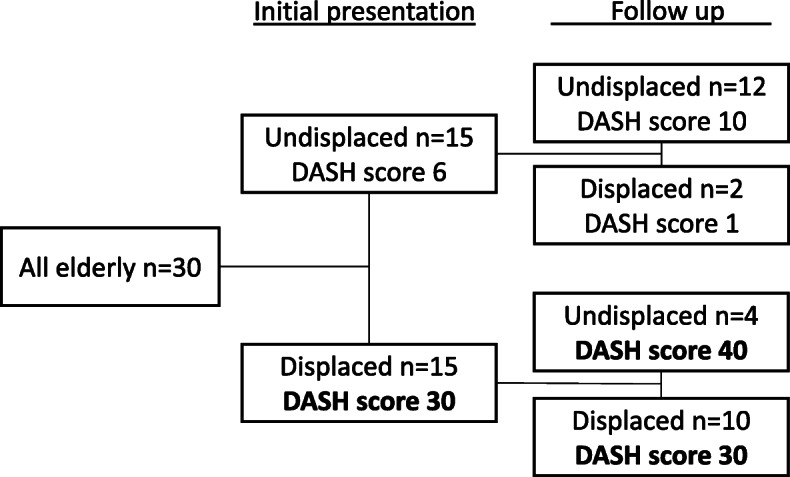
Flow chart illustrating older men and their fracture staus at initial presentation, at follow up and the median DASH* score at 1 year showing the higher DASH score in those with displaced fractures. *DASH > 15 is considered poor outcome [6]. Due to low numbers statistical significance was not tested

Fracture displacement was associated with higher median DASH scores both initially and at follow-up (undisplaced 3 vs displaced 26). Among those with undisplaced fractures, DASH scores were higher at 12 months in older men (Table 2), while age made no difference when the fracture was displaced. At radiographic follow-up, fracture displacement was not associated with DASH score in older men (*p* = 0.466), however, older men with a displaced fracture at initial presentation had higher DASH score at follow up (Fig. 4). In the younger men, there was a tendency towards worse outcome when the fracture was displaced (*p* = 0.089).

**Table 2:** Patient-related outcome by DASH-score at 1 year in younger and older men with distal radius fracture based on displacement status at follow-up

	DASH 1 year				
	n	Mean	Median (IQR)		*p*-value^β^
No displacement^a^					
< 65 years	66	5	1	(0; 7)	0.008
≥ 65 years	17	14	10	(1; 22)	
Displacement^a^					
< 65 years	5	23	38	(1; 39)	0.951
≥ 65 years	10	31	18	(1; 73)	

^a^Displacement defined as dorsal tilt > 10° and/or ulnar variance > 2 mm. ^β^Mann-Whitney test

Using SF-36 to assess global health, older men had worse physical health compared to younger men, with lower PCS at all time-points (Table 3). There was no difference in PCS or MCS in either age group comparing those with undisplaced and displaced fractures at initial presentation or at follow-up.

**Table 3:** Patient-related outcome by SF-36 physical and mental health components in younger and older men with distal radius

	Age				
	< 65 years		≥ 65 years		*p*-value
Physical Component Scale (PCS)					
6–8 weeks	43 ± 8	(21–60)	37 ± 7	(25–47)	< 0.001
6 months	51 ± 7	(31–62)	41 ± 11	(16–56)	< 0.001
12 months	52 ± 8	(28–62)	42 ± 12	(15–57)	< 0.001
Mental Component Scale (MCS)					
6–8 weeks	51 ± 11	(14–71)	44 ± 14	(15–64)	0.022
6 months	52 ± 9	(14–62)	46 ± 13	(21–64)	0.023
12 months	52 ± 8	(14–65)	50 ± 12	(27–65)	0.789

Reported values are xx (SD) and (min-max). Reference values (DK) men < 65 PCS: 54–48 MCS: 54

Men > 65 PCS: 47–44 MCS: 57–54

### Patient-related outcome measures - bone mineral density and fracture risk

Overall, BMD did not differ in those with undisplaced or displaced fracture at follow-up (not changed by adjustment for age and CCI) and presence of osteoporosis was proportionally similar regardless of fracture displacement (21%)*.* In those with osteoporosis (*n* = 24, mean 63y (range 32–88)) median DASH at 12 months was 6(IQR 1; 23) compared to 2(IQR 0; 10) in those with osteopenia or normal BMD (*p* = 0.226). Osteoporosis did not increase risk of a poor outcome, DASH > 15 (OR 2.39, 95% CI: 0.86–6.64). Neither was BMD associated with DASH score in multiple regression analysis including age, treatment method, complications, radiographic parameters and CCI in the model. Finally, using a mixed model (generalized estimating equations analysis) neither BMD (*p* = 0.574) or displacement at follow up (*p* = 0.556) contributed to the rate of recovery in DASH-score.

To investigate if distal radius fracture can also be a ‘signal fracture’ in men, we assessed the risk of future fracture using FRAX. Overall, for the *total* cohort, the median 10-year risk of a MOF was 5.1% (IQR 2.9; 9.4).

Hypothesizing that fracture displacement is likely to be an indicator of decreased bone strength, we then compared those with undisplaced and displaced fractures. FRAX estimated risk was higher among those with displaced fracture at *follow-up* vs undisplaced (9.6% vs 6.6%; *p* = 0.047), although the difference was eliminated by adjustment for age (*p* = 0.855). Among older men, we found a tendency towards higher risk in those with displaced fracture at the *initial* presentation (14% vs 8%) despite no differences in age, CCI or BMD (Table 4). Displacement also adversely affected outcome, with higher DASH scores at both 6–8 weeks and at 12 months; the higher DASH score was independent of the fracture being undisplaced or displaced after treatment at the latest examination.

**Table 4:** Patient-, fracture- and bone related factors in older men (≥65 years) according to fracture status at initial presentation

Initial presentation	Undisplaced fracture	Displaced fracture	
	*n* = 15	*n* = 15	*p*-value
Age (years)	75 (6)	76 (5)	0.613
Charlson Comorbidity Index	2 (1;3)	2 (1;4)	0.152
No. needing surgery	0	6	
No. with major complication	0	2	
BMD femoral neck (g/cm^2^)	0.864 (0.14)	0.806 (0.11)	0.239
FRAX score (median, IQR)			
Risk osteoporotic fracture	7.9 (6.3;13)	14 (8;21)	0.067
Risk Hip fracture	2.9 (1.6;6.9)	5.7 (2.9;13)	0.093
DASH score (median, IQR)			
6–8 weeks	18 (6;33)	45 (14;73)	0.024
12 months	6 (1;22)	30 (7;74)	0.035

#### Non-respondents

Information was missing for some participants: DASH was missing at both 6–8 weeks and 12 months in 14 patients; radiographic images were not available for 12 (initial) and 8 (initial and follow-up); DXA was not measured in 15. Non-responders to DASH and SF-36 were younger (41y vs 55y), had lower BMI (23 vs 26 kg/m^2^), more likely to have a type A fracture (46% vs 27%), treated with a cast (64 vs 50%) and had no complications, compared to responders (Supplementary Table 2).

## Discussion

In this study of distal radius fracture, to our knowledge the first *exclusively* in men, we found that older men more often have worse patient-related outcome in terms of function and disability and in addition, more often have displaced fractures. Osteoporosis was not associated with poor outcome, most likely a consequence of low number of participants; nevertheless, directionally the trend seems to indicate that it confers a higher likelihood. As in women, a distal radius fracture gives an indication of risk for future fracture in men, most pronounced in those with higher age but also those with displaced fractures. Age at the time of fracture is an important indicator of outcome; men older than 65 have higher disability with half experiencing poor patient-related outcome, which is in general agreement with the available literature [11, 23].

The chief concern in managing most fractures is anatomical restoration which is assumed necessary for functional outcome. In older individuals with distal radius fracture a certain degree of remaining displacement has commonly been accepted since several studies have found radiographic appearance not to be associated with self-reported outcome [17, 18, 20], however consensus is lacking [35]. In part this is related to inappropriate methodology (including not using median DASH values for statistical analysis as recommended) and inconsistent definitions of poor outcome [17, 20]. An additional reason for disparity lies in patient selection, since predominantly including those with stable fractures reduces the discriminatory ability of DASH to reflect the effect of radiographic appearance on disability. We found that radiographic appearance at follow-up was not associated with patient-related outcome in the older men, but that the initial presentation was more important with greater disability in those with displacement at that time. Notably two thirds of their fractures were still displaced at follow-up, and why an initial dislocation might be an indication of impaired bone quality unrelated to BMD.

Unraveling whether functional outcome after distal radius fracture in older men is related to normal age-related declining function or fracture appearance and deteriorated skeletal integrity associated with age is difficult.

Our results indicate that an older man with a displaced fracture at initial presentation is at higher risk of disability regardless of treatment and radiographic appearance at follow-up. This suggests that the initial displacement when the fracture occurs is critical to the final result**.** Interestingly, even when the fracture is radiographically acceptable, older men experience a higher degree of disability compared to their younger counterparts. This might be an indication of fracture instability resulting in late displacement [36]; on the other hand, it may reflect pre-existing, normal age-related reduced function. Indeed, in a Norwegian study, population-based normative data showed an increase in DASH scores with age from a mean of 8 at age 20–59 (median 2–5) to 15 in those 60 and above (median 6–14) [37]. We acknowledge that in our study, availability of a baseline pre-injury DASH score would have been valuable. The observed difference in SF-36 PCS between younger and older men is probably also explained by expected age-related decline in physical function. It is also possible that older individuals may be more accepting of impaired wrist function than younger individuals with more active lifestyles.

While we have already shown that BMD is lower in men with distal radius fracture, the obvious question is whether this has any bearing on fracture severity and displacement. For women, low BMD appears to adversely affect fracture stability [14, 15]. In our male cohort overall, BMD per se was not related to displacement, which is contrary to some studies [4, 5]; however, the majority of older men initially presenting with a displaced fracture also had unacceptable reduction at follow-up, indicating a higher degree of fracture instability and concomitant poor bone quality. Furthermore, we also found a tendency towards higher risk for future major osteoporotic fracture among these older men with displacement. The underlying pathology might reflect a higher biological age or frailty which is not captured by BMD.

So, there is a probable long-term impact of age on fracture appearance, although in the present study, a low BMD corresponding to osteoporosis was not associated with patient-related functional outcome in the first-year post-fracture. This contrasts with postmenopausal women, where osteoporosis negatively impacted DASH-score independent of fracture appearance and comorbidities [6], although a far from universal association [38, 39]. Possibly, association is obscured in our study by the relatively low number, with larger studies required before definitive conclusions can be made.

Reality has unfortunately shown that men have unacceptable low rates of evaluation for osteoporosis [9]. The higher proportion of displaced fractures in the older men taken together with the previously reported higher prevalence of osteoporosis in this cohort, imply that men, just as women, with distal radius fracture should be evaluated with DXA and FRAX as routine for secondary fracture prevention. Our data also suggests that a change in standard treatment protocol at the department may be needed, whereby older men presenting with a displaced radius fracture should have an additional follow up radiograph beyond the standard 2 weeks post treatment. Although not at higher risk of osteoporosis per se, these individuals probably have a higher degree of impaired bone strength and greater risk of late displacement.

Limitations are acknowledged. First, is the 37 % response rate. Although there is no difference in age between participants and non-participants, selection bias in a cohort study is always a possibility. Studies in men often have lower participation rates [11, 40, 41] and ours is similar to equivalent studies, although lower than those taking a mail-based approach, focusing only on self-reported outcome [11, 41] since physical participation is more demanding than returning questionnaires. Nonetheless, there might be bias towards higher DASH scores, since non-responders to one-year evaluation were younger and with less severe fracture types, a patient category usually experiencing better functional outcome. Secondly, systematic radiographic evaluation was not performed at 12 months since the study design followed our clinical practice, whereby it is not standard to perform radiographic examinations unless warranted by patient complaints. Consequently, displacements after the 2 week follow up may remain unidentified, since they are not brought to clinical attention. Thirdly, while our study is relatively large in the context of the available literature, it may be underpowered, particularly for subgroup analysis e.g. among those with displacement at follow-up. Small numbers may preclude statistical significance and the results should be interpreted bearing this in mind. Fourth, objective measures of function, such as range of motion and grip strength, which would have been valuable, were not available.

The main strengths are the prospective design, exclusive focus on men, and combination of self-reported outcome, radiographic parameters and assessment of osteoporosis and fracture risk. We cover the entire adult age-span, which is essential to capture age related differences in men with distal radius fracture and has been neglected to date. In the statistical analyses we report median DASH scores as recommended, albeit this makes direct comparisons with studies which only report mean values difficult. A strength of our study is the age stratified analysis, which provides much needed insight into patient-related functional outcome after distal radius fracture, particularly in older men, who have been underrepresented in most studies. It is apparent that further, larger investigations are warranted, and as the starting point we advocate taking a backwards approach i.e. exploring those older men exhibiting a high degree of disability after distal radius fracture, to elucidate the underlying factors contributing to worse outcome, for example through a retrospective chart review or a prospective study.

## Conclusion

Men over the age of 65 with distal radius fracture are more likely to have post-fracture disability regardless of radiographic appearance. However, fracture displacement, indicating impaired bone strength, is also more common and associated with an increased risk of fracture within 10-years. Therefore, in addition to attaining an acceptable functional outcome for the patient, it is essential to include fracture risk assessment and bone density measurement in the management of men with distal radius fracture.

## Supplementary Information

**Additional file 1: Supplementary Table 1.** Disability at 1 year according to displacement group.

**Additional file 2: Supplementary Table 2.** Comparison of respondents and non-respondents (no 1 year DASH/and or x-ray evaluation).

## Data Availability

The datasets generated and/or analysed during the current study are not publicly available but are available from the corresponding author on request.

## Abbreviations

AO: Arbeitsgemeinshaft für Osteosynthesefragen
BMD: Bone mineral density
BMI: Body mass index
CI: Confidence Interval
CCI: Charlson comorbidity index
DASH: Disabilities of the Arm, Shoulder and Hand
DXA: Dual x-ray absorptiometry
FRAX: Fracture Risk Assessment Tool
ICC: Intraclass correlation coefficient
IQR: Inter quartile range
MCS: Mental Component Scale SF-36
MOF: Major osteoporotic fracture
OR: Odds Ratio
PA: Postero-anterior
PCS: Physical Component Scale SF-36
PROM: Patient Reported Outcome Measurement
SD: Standard deviation
WHO: World Health Organisation
