# Supplementary material for: Patient-related outcome, fracture displacement and bone mineral density following distal radius fracture in young and older men
Source: BMC Musculoskelet Disord. 2020 Dec 7;21:816. doi: 10.1186/s12891-020-03843-9 (PMC7722451; doi:10.1186/s12891-020-03843-9)
Supplement: Supplementary file 1 — Additional file 1: Supplementary Table 1. Disability at 1 year according to displacement group. [file 12891_2020_3843_MOESM1_ESM.docx]

**Supplementary table 1** Disability at 1 year according to displacement group

|  |  |  | **DASH 1 year** | |  |
| --- | --- | --- | --- | --- | --- |
| **Site of displacement** | | n | Mean | Median | (IQR) |
| No malunion | | 54 | 8 | 3 | (0;10) |
| Sagittal tilt | | 6 | 25 | 20 | (1;48) |
| Ulnar variance | | 1 | 94 | 94 | - |
| Articular gap/step off | | 31 | 5 | 1 | (1;8) |
| Combined^α^ | | 7 | 25 | 26 | (0;38) |

^α^Displacement at both dorsal tilt and ulnar variance
