# Supplementary material for: Patient-related outcome, fracture displacement and bone mineral density following distal radius fracture in young and older men
Source: BMC Musculoskelet Disord. 2020 Dec 7;21:816. doi: 10.1186/s12891-020-03843-9 (PMC7722451; doi:10.1186/s12891-020-03843-9)
Supplement: Supplementary file 2 — Additional file 2: Supplementary Table 2. Comparison of respondents and non-respondents (no 1 year DASH/and or x-ray evaluation). [file 12891_2020_3843_MOESM2_ESM.docx]

**Supplementary table 2** Comparison of respondents and non-respondents (no 1year DASH/and or x-ray evaluation).

|  | Respondents | | Non-respondents | |  |
| --- | --- | --- | --- | --- | --- |
|  | n = 100 | | n = 33 | | *p*-value |
| Age, mean (SD) years | 56 | (17) | 47 | (20) | 0.022 |
| BMI, mean (SD) (g/cm^2^) | 26.2 | (0.4) | 25.0 | (0.8) | 0.223 |
| CCI, mean (SD) | 1 | (1) | 1 | (1) | 0.848 |
| Treatment- cast^§^ | 48 | (48 %) | 20 | (61 %) | 0.266 |
| Fracture type A^§^ | 25 | (25 %) | 10 | (42 %) | 0.204 |
| Osteoporosis^§^ | 21 | (21 %) | 3 | (17 %) | 0.674 |
| ^§^Values are given as n and percentage. | | | | | |
